# Supplementary material for: The Role of Aquaculture and Capture Fisheries in Meeting Food and Nutrition Security: Testing a Nutrition-Sensitive Pond Polyculture Intervention in Rural Zambia
Source: Foods. 2022 May 4;11(9):1334. doi: 10.3390/foods11091334 (PMC9102775; doi:10.3390/foods11091334)
Supplement: Supplementary file 1 [file foods-11-01334-s001.zip › foods-1596334-supplementary.pdf]

Supplementary Figure S1 (S1): Species screening and selection process (2016–2019)

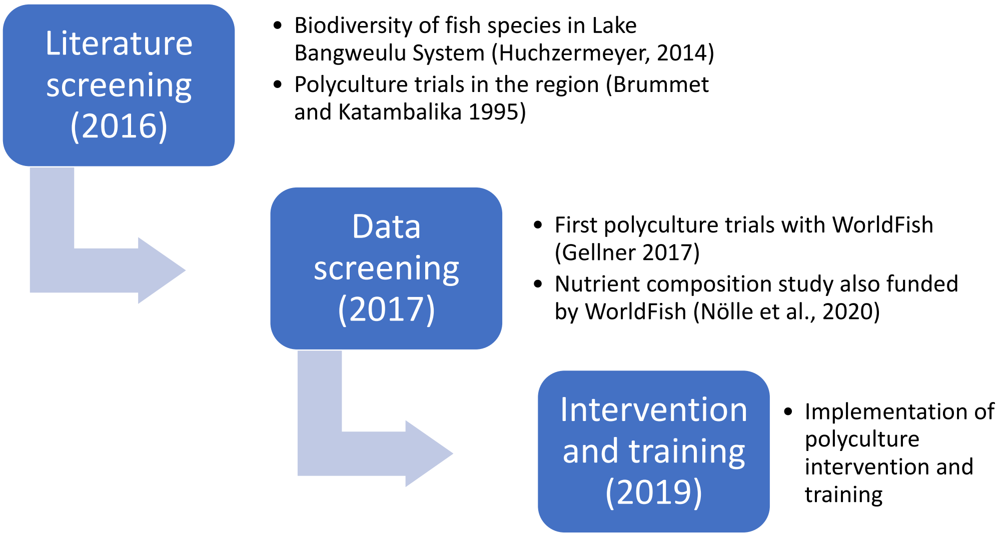

Supplementary Table S1 (S2): Recommended Nutrient Intake (RNI) for females in five age groups for 13 nutrients in grams (g)

Nutrient	Toddler 1–3 years	Children A 4–6 years	Children B 7–9 years	Adolescent 10–18 years	Adult 19–50 years
Protein (g) [1]	13	19	34	46	46
Fat (g)	29	34.8	40.6	69.6	58
n-3 fatty acids (g) [2]	1.25	1.75	1.75	3	2.5
Riboflavin (g)	0.0005	0.0006	0.0009	0.0011	0.0011
Niacin (g)	0.006	0.008	0.012	0.016	0.014
Folate (g)	0.00015	0.0002	0.0003	0.0004	0.0004
Vit_B12 (g)	0.0000009	0.0000012	0.0000018	0.0000024	0.0000024
Ca (g)	0.5	0.6	0.7	1.3	1
Fe (g) [3]	0.0058	0.0063	0.0089	0.031	0.0294
Zn (g) [4]	0.0041	0.0048	0.0056	0.0072	0.0049
K (g) [5]	2	2.3	2.3	2.3	2.6
Mg (g)	0.06	0.076	0.1	0.22	0.22
Se (g)	0.000017	0.000022	0.000021	0.000026	0.000026

[1] Based on grams per kg of body weight, e.g., for adults 0.8 g/kg body weight for the reference body weight (NASEM 2005)

[2] Calculated as 1.25 E% of female in age group, recommended intake is 0,5-2 E% and refers to the adult population (age ≥ 18 years of age): 1000 kcal for toddler, 1400 kcal for Child A, 1400 kcal for Child B, 2400 kcal, for adolescents, 2000 kcal for adults.

[3] Based on 10% bioavailability

[4] Based on Moderate bioavailability

[5] Based on Adequate Intake (AI) for potassium from NASEM (2019).

Supplementary Table S2 (S3): Nutrient composition of fish species by form (source: Nölle et al., 2020)

Species	Form from survey	Form of fish in lab	Substitute Species	Portion (100 g)	Protein (g)	Fat (g)	Water (g)	ALA (% of fat)	EPA (% of fat)	FHA (% of fat)	Riboflavin (mg)	Niacin (mg)	Folate (mcg)	Vit B12 (mcg)	Calcium (mg)	Iron (mg)	zinc (mg)	Potassium (mg)	Magnesium (mg)	Selenium (mcg)
B. trimaculatus	Fresh	Fresh	B. radiatus	whole	21.8	8.6	65.3	2.2	0.7	1.3	0.1	1.8	21.7	14.9	1324.4	6.7	5.2	282.5	56.1	43.9
	Dried	Dry	None	whole	56.2	23.6	8.9	1.5	0.4	1.7	0.1	4.9	52.4	30.6	3001.3	11.3	12.1	920.7	131.0	100.7
	Smoked	Dry	None	whole	56.2	23.6	8.9	1.5	0.4	1.7	0.1	4.9	52.4	30.6	3001.3	11.3	12.1	920.7	131.0	100.7
Catfish	Fresh	Fresh	None	filleted	16.5	1.4	81.4	2.5	0.4	2.9	0.1	2.9	20.7	3.9	10.2	0.7	0.7	296.3	21.5	36.4
	Dried	Dry	None	filleted	67.9	19.1	9.1	3.3	1.1	2.0	0.2	8.4	48.9	33.8	482.8	9.0	3.1	1243.5	104.7	222.4
	Smoked	Smoked	None	filleted	29.8	2.8	66.8	1.5	0.2	2.7	0.1	4.3	21.6	5.3	15.1	13.7	1.5	500.0	29.5	43.0
L. miodon	Fresh	Fresh	None	gutted	16.5	1.8	77.6	2.4	2.8	7.8	0.1	3.6	14.1	9.7	823.8	2.9	3.7	340.4	41.6	35.4
	Dried	Dry	None	whole	67.5	11.0	9.0	3.4	3.9	7.8	0.4	20.7	63.0	41.1	2713.8	9.1	13.4	1421.3	162.7	158.5
	Smoked	Dry	None	whole	67.5	11.0	9.0	3.4	3.9	7.8	0.4	20.7	63.0	41.1	2713.8	9.1	13.4	1421.3	162.7	158.5
L. stappersii	Fresh	Fresh	None	filleted	23.2	2.4	73.7	0.9	0.7	2.6	0.1	7.6	14.0	5.6	30.1	0.8	0.6	326.4	28.1	128.2
	Dried	Smoked	M. Lacerde	body	64.1	22.8	9.8	2.7	0.7	6.9	0.3	4.0	44.0	3.8	1169.3	3.7	5.0	806.1	84.1	107.7
	Smoked	Smoked	M. Lacerde	body	64.1	22.8	9.8	2.7	0.7	6.9	0.3	4.0	44.0	3.8	1169.3	3.7	5.0	806.1	84.1	107.7
M. longirostris	Fresh	Fresh	None	filleted	13.6	2.5	83.4	1.1	1.6	2.4	0.1	0.5	5.6	2.6	37.2	0.5	0.4	159.5	16.7	25.4
	Dried	Smoked	M. Lacerde	body	64.1	22.8	9.8	2.7	0.7	6.9	0.3	4.0	44.0	3.8	1169.3	3.7	5.0	806.1	84.1	107.7
	Smoked	Smoked	M. Lacerde	body	64.1	22.8	9.8	2.7	0.7	6.9	0.3	4.0	44.0	3.8	1169.3	3.7	5.0	806.1	84.1	107.7
M. macrolepidotus	Fresh	Fresh	None	filleted	17.1	8.1	71.9	2.5	0.6	1.1	0.4	1.3	7.4	4.8	692.2	1.2	3.4	291.0	35.7	34.0
	Dried	Dry	None	gutted	66.0	12.1	10.5	3.5	1.0	2.2	0.7	4.9	84.1	52.8	2882.5	20.0	13.8	1156.5	151.8	149.4
	Smoked	Dry	None	gutted	66.0	12.1	10.5	3.5	1.0	2.2	0.7	4.9	84.1	52.8	2882.5	20.0	13.8	1156.5	151.8	149.4
O. macrochir	Fresh	Fresh	None	filleted	18.4	1.4	79.3	1.7	0.2	2.3	0.1	4.0	6.0	2.3	41.9	0.4	1.2	335.1	23.6	19.5
	Smoked	Smoked	T. Rendalli	body	67.3	10.5	19.7	1.4	0.0	0.1	0.7	12.5	33.7	4.3	323.1	1.8	4.9	1116.8	84.3	105.6
	Dried	Dry	T. sparmanii	whole	57.3	19.7	7.7	5.2	0.5	1.9	1.0	6.6	42.2	29.6	3463.8	38.0	9.4	1083.7	136.9	44.5
P. acutirosis	Fresh	Fresh	L. miodon	whole	16.5	1.8	77.6	2.4	2.8	7.8	0.1	3.6	14.1	9.7	823.8	2.9	3.7	340.4	41.6	35.4

	Dried	Dry	None	whole	67.9	12.7	7.3	3.4	3.7	8.6	0.2	7.3	53.3	25.0	2975.3	22.1	19.3	1325.5	171.2	106.5
	Smoked	Dry	None	whole	67.9	12.7	7.3	3.4	3.7	8.6	0.2	7.3	53.3	25.0	2975.3	22.1	19.3	1325.5	171.2	106.5
P. philander	Fresh	Fresh	T. Rendalli / sparrmanii	filleted	19.7	1.2	78.6	4.1	0.6	3.0	0.1	2.3	9.2	1.5	54.9	0.4	1.0	226.1	21.5	132.2
	Dried	Dry	None	whole	57.0	17.3	8.7	2.7	1.3	4.4	0.8	8.1	31.1	9.2	4361.0	17.3	10.9	1134.8	168.7	65.7
	Smoked	Dry	None	whole	57.0	17.3	8.7	2.7	1.3	4.4	0.8	8.1	31.1	9.2	4361.0	17.3	10.9	1134.8	168.7	65.7
S. angusticeps	Fresh	Fresh	None	filleted	17.7	0.8	80.9	1.1	0.2	2.7	0.1	1.6	23.3	0.9	19.9	0.2	0.6	314.1	24.2	23.9
	Dried	Smoked	S. Robustus	body	76.9	6.8	13.5	1.9	0.3	6.2	0.3	6.9	27.6	8.6	527.5	1.6	3.2	1128.2	96.1	99.8
	Smoked	Smoked	S. Robustus	body	76.9	6.8	13.5	1.9	0.3	6.2	0.3	6.9	27.6	8.6	527.5	1.6	3.2	1128.2	96.1	99.8
S. mellandi	Fresh	Fresh	None	whole	18.0	4.2	73.9	2.6	0.8	2.5	0.2	2.2	27.1	4.5	1028.4	3.0	2.2	260.5	42.8	38.8
	Dried	Smoked	None	body	70.4	15.6	12.0	1.1	0.2	0.8	0.8	9.3	46.7	12.1	899.1	3.0	4.6	1081.7	102.0	116.3
	Smoked	Smoked	None	body	70.4	15.6	12.0	1.1	0.2	0.8	0.8	9.3	46.7	12.1	899.1	3.0	4.6	1081.7	102.0	116.3
S. mystus	Fresh	Fresh	A. occidentalis	filleted	17.0	1.1	81.5	2.0	1.5	2.3	0.1	3.1	19.2	2.5	13.7	0.4	0.4	280.8	20.3	15.2
	Dried	Dry	S. intermedius	body	59.6	9.5	10.4	4.0	1.3	4.5	0.2	6.4	125.0	24.0	2781.2	9.1	6.6	1157.2	142.9	143.8
	Smoked	Smoked	S. intermedius	body	62.0	20.8	13.7	7.8	1.3	2.3	0.2	4.2	33.3	15.8	978.1	7.7	4.1	1084.4	116.6	103.4
Synodontis spp	Fresh	Fresh	None	whole	16.5	14.4	67.9	1.1	0.2	0.2	0.1	2.7	13.5	3.7	26.8	0.6	0.5	189.9	16.2	76.9
	Dried	Smoked	None	body	62.9	28.0	8.2	2.8	0.9	2.6	0.1	7.7	19.0	8.4	304.8	7.2	2.8	806.3	70.6	111.1
	Smoked	Smoked	None	body	62.9	28.0	8.2	2.8	0.9	2.6	0.1	7.7	19.0	8.4	304.8	7.2	2.8	806.3	70.6	111.1
T. rendalli	Fresh	Fresh	None	filleted	19.7	1.2	78.6	4.1	0.6	3.0	0.2	3.6	11.7	1.0	58.6	0.4	1.1	333.5	26.6	23.6
	Dried	Dry	T. sparrmanii	whole	59.7	14.0	9.0	3.9	0.3	1.0	0.8	8.1	31.1	9.2	4225.8	70.1	10.8	991.5	150.6	139.2
	Smoked	Smoked	None	body	67.3	10.5	19.7	1.4	0.0	0.1	0.7	12.5	33.7	4.3	323.1	1.8	4.9	1116.8	84.3	105.6
T. sparrmanii T	Fresh	Fresh	T. Rendalli / sparrmanii	filleted	19.7	1.2	78.6	4.1	0.6	3.0	0.1	2.3	9.2	1.5	54.9	0.4	1.0	226.1	21.5	132.2
	Dried	Dry	None	whole	59.7	14.0	9.0	3.9	0.3	1.0	0.8	8.1	31.1	9.2	4225.8	70.1	10.8	991.5	150.6	139.2
	Smoked	Dry	None	whole	59.7	14.0	9.0	3.9	0.3	1.0	0.8	8.1	31.1	9.2	4225.8	70.1	10.8	991.5	150.6	139.2

Supplementary Table S3 (S4): Total frequency of species that were combined into one species

Species	Freq.	Combined species	Freq.	New total
C. stappersii	7	Catfish	458	465
C. multispine	26	Synodontis	53	79
R. argentea	4	L. miodon	133	137
P. mueruensis	26	P. acutirosis	107	133
S. robustus	10	S. mellandi	79	89
T. ruweti	10	T. sparrmanii	543	553
